# Severe Primary Hyperparathyroidism Caused by Parathyroid Carcinoma in a 13‐Year‐Old Child; Novel Findings From HRpQCT

**DOI:** 10.1002/jbm4.10324

**Published:** 2020-01-02

**Authors:** Nina Lenherr‐Taube, Carol KL Lam, Reza Vali, Amer Shammas, Paolo Campisi, Faisal Zawawi, Gino R Somers, Jennifer Stimec, Ozgur Mete, Andy KO Wong, Etienne Sochett

**Affiliations:** ^1^ Department of Pediatrics, Division of Endocrinology Toronto Canada; ^2^ University of Toronto Toronto Canada; ^3^ Department of Diagnostic Imaging, Division of Nuclear Medicine Hospital for Sick Children Toronto Canada; ^4^ Department of Otolaryngology – Head & Neck Surgery Hospital for Sick Children Toronto Canada; ^5^ Department of Laboratory Medicine & Pathology Hospital for Sick Children Toronto Canada; ^6^ Department of Diagnostic Imaging Hospital for Sick Children Toronto Canada; ^7^ Department of Pathology University Health Network, Princess Margaret Cancer Centre Toronto Canada; ^8^ Joint Department of Medical Imaging, Toronto General Research Institute University Health Network Toronto Canada; ^9^ Department of Epidemiology Dalla Lana School of Public Health Toronto Canada

**Keywords:** ANTIRESORPTIVES, BONE QCT/UCT, PARATHYROID‐RELATED DISORDERS, RADIOLOGY, TUMOR‐INDUCED BONE DISEASE

## Abstract

Primary hyperparathyroidism is a condition that occurs infrequently in children. Parathyroid carcinoma, as the underlying cause of hyperparathyroidism in this age group, is extraordinarily rare, with only a few cases reported in the literature. We present a 13‐year‐old boy with musculoskeletal pain who was found to have brown tumors from primary hyperparathyroidism caused by parafibromin‐immunodeficient parathyroid carcinoma. Our patient had no clinical, biochemical, or radiographic evidence of pituitary adenomas, pancreatic tumors, thyroid tumors, pheochromocytoma, jaw tumors, renal abnormalities, or testicular lesions. Germline testing for *AP2S1*, *CASR*, *CDC73/HRPT2*, *CDKN1B*, *GNA11*, *MEN1, PTH1R*, *RET,* and the *GCM2* gene showed no pathological variants, and a microarray of *CDC73/HRPT2* did not reveal deletion or duplication. He was managed with i.v. fluids, calcitonin, pamidronate, and denosumab prior to surgery to stabilize hypercalcemia. After removal of a single parathyroid tumor, he developed severe hungry bone syndrome and required 3 weeks of continuous i.v. calcium infusion, in addition to oral calcium and activated vitamin D. Histopathological examination identified an angioinvasive parathyroid carcinoma with global loss of parafibromin (protein encoded by *CDC73/HRPT2*)*.*HRpQCT and DXA studies were obtained prior to surgery and 18‐months postsurgery. HRpQCT showed a resolution of osteolytic lesions combined with structural improvement of cortical porosity and an increase in both cortical thickness and density compared with levels prior to treatment. These findings highlight the added value of HRpQCT in primary hyperparathyroidism. In addition to our case, we have provided a review of the published cases of parathyroid cancer in children. © 2019 The Authors. *JBMR Plus* published by Wiley Periodicals, Inc. on behalf of American Society for Bone and Mineral Research.

## Introduction

Primary hyperparathyroidism (PHPT) is a rare condition in children that is often associated with mutations in *MEN1*, *RET*, *CDKN1B*, *PRAD1*, or *CDC73/HRPT2*. Parathyroid adenoma is found in approximately 80% of pediatric and adult patients with PHPT. Parathyroid carcinoma accounts for less than 1% of cases, almost all of whom are adults.^1^, ^2^ The morphological diagnosis of parathyroid carcinoma can be rendered when a parathyroid neoplasm exhibits any of the following: (i) angioinvasion (vascular invasion, often venous), (ii) lymphatic invasion, (iii) perineural invasion, (iv) local gross malignant invasive growth into adjacent organs or surrounding tissues, or (v) metastatic spread.^3^ Several biomarkers can also assist this diagnosis.^4^, ^5^, ^6^, ^7^ Treatment of PHPT in children is primarily surgical, following correction of the serum calcium and any fluid deficits. More recent reports have stressed the importance of distinguishing parathyroid adenoma from carcinoma given that the surgical approach to parathyroid carcinoma is primarily en bloc resection by an experienced surgeon.^8^, ^9^ Studies have shown that long‐term survival is around 89% following en bloc resection, compared with 53% in patients following standard parathyroidectomy.^10^ Radiation and chemotherapy have been used as adjuvant therapies for metastases, but are generally not curative.^11^ The preoperative recognition of parathyroid carcinoma is challenging in the patient with PHPT. Parathyroid carcinoma should be suspected with severe PTH‐dependent hypercalcemia and its complications. When a carcinoma is under consideration, it is important to alert the surgeon of this possibility and engage a histopathologist who is expert in parathyroid pathology.^2^

Around 80% of sporadic parathyroid carcinomas harbor inactivating *CDC73/HRPT2* mutations, leading to global loss of parafibromin expression in the tumor.^7^ Although around 20% of sporadic‐appearing parathyroid carcinomas may be related to underlying *CDC73/HRPT2* mutations, germline *CDC73/HRPT2* mutations have also been described in familial isolated hyperparathyroidism (FIHP; OMIM #145000) and hyperparathyroidism‐jaw tumor syndrome (HP‐JT; OMIM #145001).^12^, ^13^ These typically manifest with parathyroid neoplasms with a lifetime increased risk of parathyroid carcinoma.^14^, ^15^, ^16^

We present here the case of a 13‐year‐old boy who presented with musculoskeletal pain and brown tumors, and was found to have PHPT caused by sporadic parafibromin‐immunodeficient pararthyroid carcinoma. HRpQCT scans were obtained before and 1.5 years following the surgery and provide insights into the effect of PHPT on bone not observed from DXA. Intravenous bisphosphonate supplemented by denosumab was required to normalize the serum calcium preoperatively. Given the extreme rarity of this condition, a literature review of pediatric parathyroid carcinoma cases is provided in Supplemental Table S1.

## Clinical Vignette

**Figure 1:**
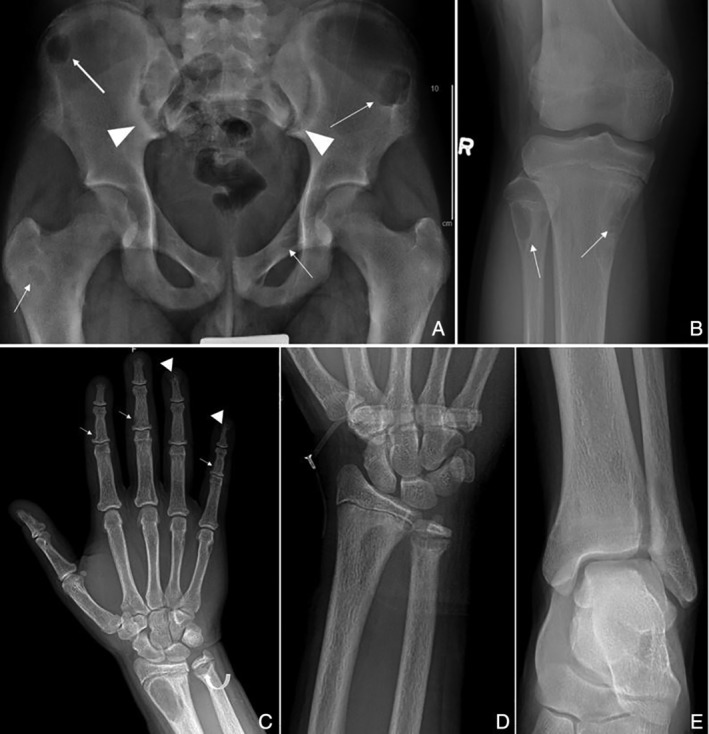
Anteroposterior (AP) radiograph of the pelvis (*A*) and right knee (*B*): Multiple well‐defined, lytic lesions (arrows) seen throughout the pelvis and proximal tibia and fibula with thin sclerotic borders compatible with brown tumors. Subchondral resorption at the sacroiliac joints with widening and adjacent sclerosis is more pronounced on the iliac side (arrowheads). AP radiograph right hand (*C*): Subperiosteal resorption along the radial aspects of the middle phalanges (arrows), and resorption of the distal phalangeal tufts consistent with acro‐osteolysis (arrowheads). Subphyseal resorption along the distal ulnar metaphysis resulting in physeal widening (curved arrow). AP radiograph of the left wrist (*D*): Site of HRpQCT distal radius scan, and left ankle (*E*): Site of HRpQCT distal tibia scan.

A previously healthy 13‐year‐old boy was initially seen at a sports medicine clinic for significant bilateral genu varum and signs of hip impingement on examination. Bilateral hip and knee X‐rays were taken and showed multiple bony lytic lesions throughout the skeleton and widening of the bilateral sacroiliac joints (Fig. 1 *A–E*). The patient was referred to our tertiary care for a whole‐body MRI because of concerns for potential malignancy, or alternatively, that the lytic lesions might be metastases. Imaging revealed multifocal lesions throughout the axial and appendicular skeleton in association with areas of subperiosteal resorption and sacroiliac joint widening. In addition, a well‐defined 2.3‐ × 1.5‐cm short tau inversion recovery hyperintense lesion in the right pretracheal region, suggestive of a parathyroid adenoma, was noted. A biochemical evaluation was obtained and showed hypercalcemia caused by PHPT (calcium 3.85 mmol/L, PTH 980 ng/L). The patient was admitted to our hospital for management of the severe hypercalcemia and further workup (Table 1). A subsequent neck ultrasound confirmed a heterogeneous, well‐defined, noncalcified solid lesion measuring 2.3 × 1.3 × 1.6 cm. A technetium‐99m sestamibi scan showed a persistent small focus of prominent tracer retention within the right lower thyroid lobe, suggesting a single parathyroid adenoma.

**Table 1:** Laboratory Findings and Imaging at Diagnosis and Approximately 1‐Year Postsurgery

	Reference range	Initial presentation	~One year post‐surgery
Biochemistry			
Calcium (mmol/L)	2.22–2.54	3.85	2.45
Ionized calcium (mmol/L)	1.22–1.37	2.12	1.26
Phosphate (mmol/L)	1.18–1.98	0.68	1.36
PTH (ng/L)	12–78	980	29
Alkaline phosphatase (μkat/L) [U/L]	2.07–7.92 [124–474]	20.79 [1245]	1.85 [111]
25OHD (nmol/L)		49	78
Calcium:creatinine ratio urine (mmol/mmol)	<0.6	2.3	0.36
TSH (mU/L)	0.73–4.09	1.06	1.54
Free T4 (pmol/L)	10.0–17.6	10.9	11.8
Imaging			
X‐ray knees and hips (2 views)		Multiple lytic lesions Subchondral resorption at SI joints.	Healing of multiple lucent osseous lesions. Resolution of SI joint widening.
MRI whole body w/o contrast		Multifocal lesions throughout skeleton. Nonspecific lesion of the right parathyroid.	Interval decrease in size or resolution of multiple bone lesions, no new lesions.
BMD			
‐ (L1–L4) *Z*‐score		1.2	1.8
‐ TBLH (g/cm^2^)		0.970	1.233
‐ AP Spine BMD (g/cm^2^)		1.099	1.320
HRpQCT		See Table 2	See Table 2

SI = sacroiliac; TBLH = total body less head; AP = anteroposterior.

On history, the patient was born to a nonconsanguineous Southeast Asian couple. Family history revealed a paternal uncle who died in middle‐age from a jaw mass that was reported to have resulted from leukemia. He had experienced mild polyuria, polydipsia, and lower leg pain, but no other significant signs and symptoms of severe hypercalcemia. On further workup, there was no clinical, biochemical, or radiographic evidence of pituitary adenomas, pancreatic tumors, thyroid tumors, pheochromocytoma, jaw tumors, renal abnormalities, or testicular lesions. Whole‐blood genetic sequencing for *AP2S1*, *CASR*, *CDC73*, *CDKN1B*, *GNA11*, *MEN1*, *PTH1R*, *RET*, and the *GCM2* gene showed no pathological variants, and microarray of *CDC73/HRPT2* did not reveal deletions or duplications.

A radiographic skeletal survey revealed numerous lucent lesions compatible with brown tumors. Additional areas of subchondral bone resorption were identified at the distal clavicles, and subphyseal resorption within the remaining patent growth plates of the upper and lower extremities. Subperiosteal resorption in the classic location along the radial aspect of the middle phalanges of the hands, and acro‐osteolysis of the distal tufts, highly suggestive of hyperparathyroidism (Fig. 1 *C*), were observed.

**Figure 2:**
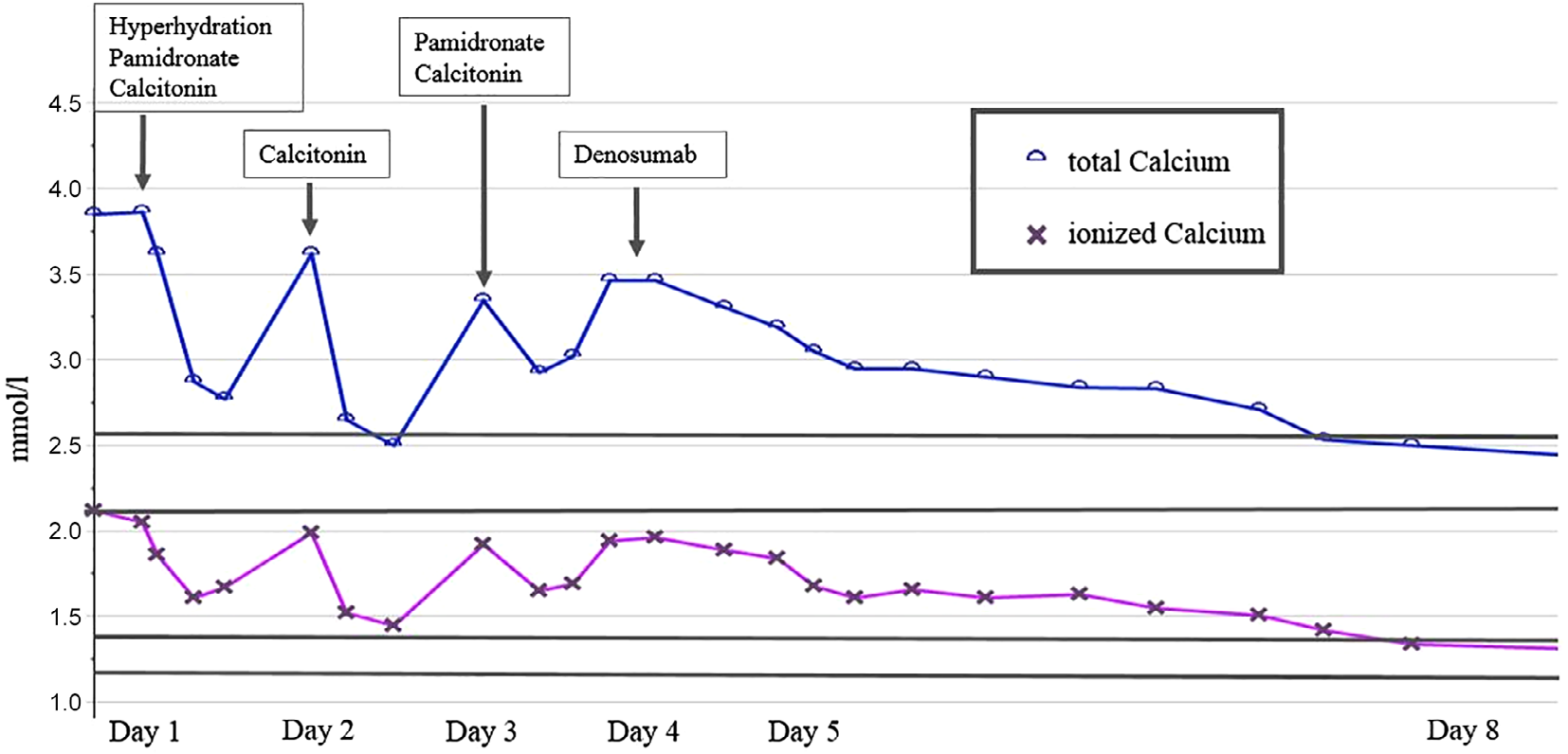
Overview of preoperative management of severe hypercalcemia during 8 days of admission prior to surgery.

Given the very high serum calcium level, and with the plan for referral to surgery, he was managed initially with i.v. hydration with normal saline, two doses of s.c. calcitonin (4 U/kg = 320 U) in conjunction with the first dose of pamidronate (0.5 mg/kg). A second dose of pamidronate was required, but failed to reduce the serum calcium sufficiently to permit surgery. Subsequently, one dose of subcutaneous denosumab (60 mg) was administered on day 4; thereafter, the serum calcium level decreased and normalized by day 8 of admission (Fig. 2).

**Figure 3:**
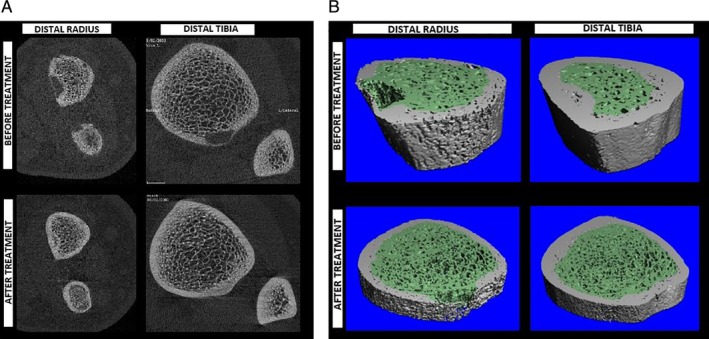
Comparison of distal radius and distal tibia bone quality before and after treatment within (*A*) 2D transaxial slices and (*B*) 3D renderings of CT segmentations. Both the distal radius and tibia showed compaction of trabecular bone with tighter separation between trabeculae. The distal radius showed more cortical porosity before treatment, which resolved along with the trabecular compaction to some degree after therapy. Regularity of the trabecular distribution is shown to have improved following therapy. Osteolytic lesions are apparent in both 2D and 3D representations, and the resolution after surgery and treatment is notable.

Further musculoskeletal imaging studies were performed during this admission and prior to surgery. BMD using DXA scans (GE Lunar Prodigy Advance; GE Lunar, Madison, WI, USA) showed a lumbar spine (L1 to L4) *Z*‐score of 1.2. HRpQCT scans (XtremeCT II; Scanco Medical AG, Brüttisellen, Switzerland) of the distal tibia and distal radius were performed using a standard protocol (62‐μm isotropic voxel size) with scans prescribed at 8% of the tibial length (medial malleolus to medial tibial plateau) measured proximally from the tibial endplate; and 7% of the radial length (base of olecranon process to styloid process of the ulna) measured proximally from the radial inclination as recommended for children by Burrows and colleagues.^18^ These specialized CT scans were compared with age‐ and sex‐matched healthy controls. At this presurgical stage, scans showed a significantly lower number of trabeculae and higher cortical porosity at the distal tibial site, but other features were within range for our healthy controls (Table 2). At the distal radius, the trabecular number was also lower, albeit not as reduced as at the distal tibia, and cortical bone density was significantly lower, associated with much greater cortical porosity versus the healthy norms. At both sites, the thickness of the trabeculae was greater and total bone density was normal or in a higher range—this could explain the normal BMD *Z*‐score from DXA (1.2), despite aforementioned structural abnormalities. It is also notable that the cortex, despite being porous and less dense, was much thicker than normal—a phenomenon that could be explained by periosteal expansion to accommodate for mechanical weakness (Table 2). Upon inspection of the 3D models of these CT images and their corresponding transaxial slices, osteolytic lesions were notable in both the distal radius and tibia scans, mostly occupying the cortical compartment, and would easily explain the apparent thickening of the cortex and concurrent loss of cortical density (Fig. 3 *A*,*B*). These lesions were consistent with those observed on the skeletal survey at the wrist and ankle (Fig. 1 *D*,*E*).

**Table 2:** Comparison of HRpQCT v2 Parameters Before and After Treatment and to Age‐ and Sex‐Matched Norms

Distal tibia		8% site				
site	Baseline	follow‐up	Norm		% Change	% LSC
			Mean	Lower CI, upper CI		
tt.vBMD	327.8	464.2	272.4	200.7, 344.0	**41.61**	4.7
tb.vBMD	237.4	294.4	232.6	171.2, 294.0	**24.01**	5.0
BV/TV	0.367	0.431	0.345	0.243, 0.447	**17.44**	4.9
Tb.N	1.4	1.8	1.9	1.6, 2.2	**28.57**	15.0
Tb.Th	0.327	0.368	0.248	0.211, 0.285	12.54	13.7
Ct.vBMD	619.9	774.7	677.0	576.2, 777.9	**24.97**	3.5
Ct.Th	1.679	3.109	1.011	0.521, 1.501	**85.17**	17.9
Ct.Po	7.9	3.9	2.9	0.5, 5.3	**−50.63**	9.1

Distal radius		7% site				
Site	Baseline	Follow‐up	Norm		% Change	% LSC
			Mean	Lower CI, upper CI		
tt.vBMD	440.1	639.1	285.9	199.3, 372.6	**45.22**	7.2
tb.vBMD	280.2	320.8	225.8	155.6, 295.9	**14.49**	3.9
BV/TV	0.424	0.465	0.329	0.213, 0.446	**9.67**	3.7
Tb.N	1.6	1.8	1.8	1.5, 2.0	14.38	17.0
Tb.Th	0.326	0.357	0.246	0.203, 0.290	9.51	15.3
Ct.vBMD	600.5	834.6	655.9	606.1, 705.8	**38.98**	7.6
Ct.Th	2.012	3.040	0.876	0.483, 1.269	**51.09**	40.0
Ct.Po	10.5	4.4	1.9	0.9, 3.0	**−58.10**	27.6

Baseline and follow‐up values were positioned within above or below reference ranges determined from healthy controls expressed as 95% confidence interval (CI) around the mean. Percent least significant change (% LSC) values, representing the minimal change required to be considered clinically significant, were referenced from Kawalilak and colleague.^17^ Boldface indicates clinically significant change in the positive direction towards improved bone quality.

tt.vBMD = total volumetric BMD; tb.vBMD = trabecular volumetric BMD; BV/TV = bone volume/total volume; Tb.N = trabecular number; Tb.Th = trabecular thickness; Ct.vBMD = cortical volumetric BMD; Ct.Th = cortical thickness; Ct.Po = cortical porosity.

On day 12 of admission, the patient underwent surgical parathyroidectomy, with removal of the right inferior parathyroid gland and complete excision of the lesion. A downward trend of the intraoperative PTH level was achieved (preoperative PTH level: 2906 ng/L, intraoperative PTH level: 16 ng/L), indicating successful removal of the hyperfunctioning lesion. Postoperatively, the patient was closely monitored and managed for hungry bone syndrome. Oral calcium carbonate and calcitriol was initiated postsurgery once he was able to tolerate fluids. An i.v. calcium infusion was started at a serum calcium of 1.93 mmol/L, and titrated to target a calcium level in the reference range: 0.01 to 0.02 mmol elemental calcium/kg/hour. The calcium infusion was weaned and by 3 weeks postsurgery, he was able to maintain normocalcemia with oral calcium carbonate (2000 mg four times a day) and calcitriol (0.03 μg/kg twice a day) alone. After discharge, serum calcium and PTH levels were monitored frequently and adjustments made based on the acquired levels. The calcium carbonate and calcitriol were discontinued 10 months after surgery.

**Figure 4:**
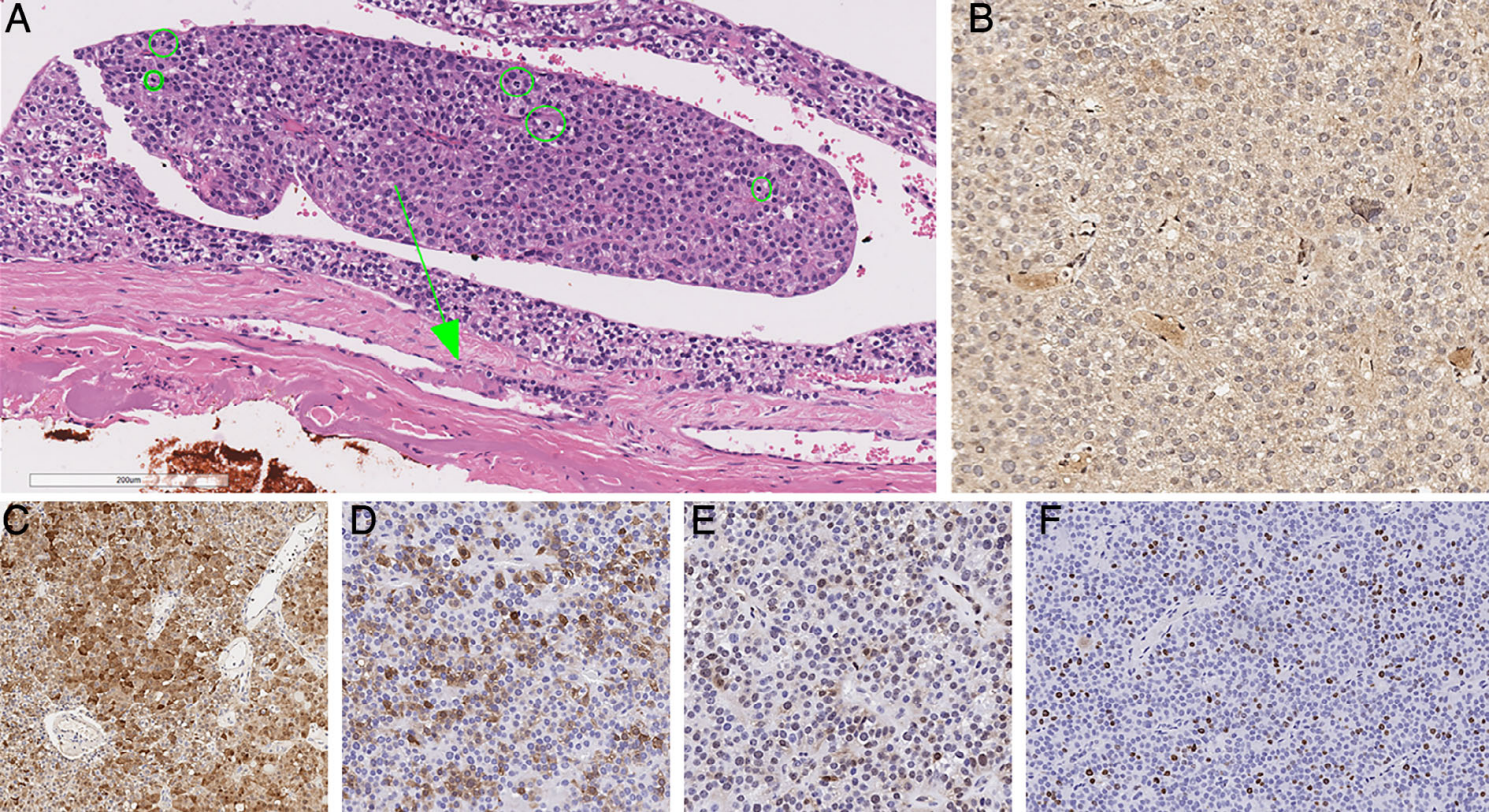
Histopathology of parathyroid carcinoma. The tumor showed increased mitotic activity as well as a focus of angioinvasion (arrows) characterized by intravascular tumor cells admixed with thrombus (*A*). Green circles highlight mitotic figures in this photomicrograph (*A*). The tumor showed global loss of parafibromin expression, while the endothelial cells remained positive (*B*). The tumor cells were also variably positive for PGP9.5 (*C*) and galectin‐3 (*D*), and showed reduced expression for p27 (*E*). While the tumor displayed intratumoral proliferative heterogeneity, the MIB‐1 labeling index was as high as 25.45% in hot spots (*F*).

Histopathological examination of the parathyroidectomy specimen showed a parathyroid neoplasm with nests and sheets of polygonal cells with pale to clear cytoplasm and round central nuclei with stippled chromatin and occasional small basophilic nucleoli (Fig. 4). Also present were clusters of oncocytes with prominent amounts of eosinophilic cytoplasm as well as cells with larger nuclei, occasional bizarre hyperchromatic nuclei, and increased proliferative activity (Fig. 4 *A*). The mitotic activity was 16.6 per 10 high‐power fields (HPF), based on 83 per 50 HPF from high mitotic density areas using a phosphohistone‐H3‐assisted mitotic count. Occasional atypical mitoses were also seen. The tumor was surrounded by a thin fibrous capsule, and there was evidence of angioinvasion characterized by intravascular tumor cells admixed with thrombus (Fig. 4 *A*). Parafibromin (protein encoded by *CDC73/HRPT2*) immunohistochemistry showed a global loss of nuclear and nucleolar staining, whereas the internal control (eg, endothelial cells) remained positive for parafibromin (Fig. 4 *B*). The tumor was positive for PGP9.5 (Fig. 4 *C*) and galectin‐3 (Fig. 4 *D*), and showed reduced expression for bcl‐2 and p27 (Fig. 4 *E*). There was also focal reduction in allophycocyanin staining. The MIB‐1 labeling index was 25.45% in 9588 tumor cells from hot spots using a Leica Biosystems (Leica, Wetzlar, Germany) automated image‐analysis nuclear algorithm (Fig. 4 *F*). The overall morphological and immunohistochemical findings were those of a parathyroid carcinoma. Given the age of the patient, menin (protein encoded by *MEN1*) immunohistochemistry was also performed. The tumor showed no loss of menin expression making *MEN1*‐driven pathogenesis unlikely. As the tumor was completed resected, our patient did not require adjuvant therapy. Genetic studies for possible genetic forms of parathyroid carcinoma (multiple endocrine neoplasia types 1, 2, and 4,^19^ *CDC73/HRTP2*‐driven familial isolated PHPT, and HP‐JT syndrome) were negative.

Repeat imaging studies using HRpQCT at 1.5 years following surgery showed significant improvement for most measured cortical and trabecular features, including higher volumetric density, and greater fraction of trabecular bone along with a larger number of trabeculae balanced by thicker and denser cortices. These improvements were benchmarked against the least significant change (LSC) measure previously reported by Kawalilak and colleagues,^17^ which is meant to reflect the minimal amount of change to be considered clinically meaningful. The greatest changes appeared to reside in the cortices at both the distal radius and tibia sites (Table 2). In fact, when compared with our age‐ and sex‐matched norms, postsurgical values were well above healthy control levels. Upon inspection of both 2D transaxial slices (Fig. 3 *A*) and corresponding 3D CT models (Fig. 3 *B*), it was noticed that the osteolytic lesions were resolved by new bone formation. Whole‐body MRI supported these observations showing a decrease in size or complete resolution of multiple bone lesions with no new lesions present.

## Discussion

PHPT is uncommon in the pediatric population, and parathyroid carcinoma is an extremely rare cause of PHPT. A review of the literature identified only 12 other pediatric cases (Supplementary Table S1).^8^, ^20^, ^21^, ^22^, ^23^, ^24^, ^25^, ^26^, ^27^, ^28^, ^29^ Demographic details appear to match those of our patient with the median age of presentation reported to be 13 years of age and a near 1:1 male‐to‐female ratio. Of the eight cases in the literature with follow‐up and relapse data, 3 patients appear to have relapsed with pulmonary metastases within a timeframe of 6 months to 5 years. Our patient had a solitary parathyroidectomy with clear pathological margins and had no signs of relapse at 18‐month follow‐up.

There are a number of features to our case that warrant highlighting. First, successful resolution of the severe hypercalcemia required denosumab in addition to the standard treatment of hypercalcemia with fluid hyperhydration, short‐term calcitonin, and i.v. bisphosphonate. Denosumab is a human monoclonal antibody that binds with high affinity to RANKL. RANKL is increased in PHPT and the binding of RANKL by denosumab reduces osteoclastic bone resorption. Indeed, in our case, one dose of denosumab following pamidronate (total dose 1.0 mg/kg) rapidly achieved normalization of hypercalcemia. Given the severity of the initial presentation, hungry bone syndrome was anticipated despite the use of bisphosphonates, which have been implicated to reduce the risk of this postsurgical complication in patients with hyperparathyroidism.^30^ An interesting consideration in the use of denosumab, given its half‐life of 30 days^31^ and mechanism of action, is whether it might prolong postsurgical hypocalcemia. The duration and requirement for i.v. calcium in our patient were similar to other cases of parathyroid carcinoma described in the literature.^23^, ^26^ Nonetheless, we cannot exclude the possibility that denosumab may have contributed to the need for i.v. calcium support.

Second, the role of pathologists in the workup of parathyroid carcinomas has evolved over the past decade: A recent consensus statement suggested that parathyroid carcinoma should be considered in severe PTH‐dependent hypercalcemia and that in these cases histopathology should be performed by an expert parathyroid pathologist to distinguish parathyroid carcinoma from adenoma in a standardized fashion.^2^ Our patient presented with significant hypercalcemia and brown tumors as indicators of severe PHPT; therefore, a detailed histopathologic workup was indicated. Although the diagnosis of parathyroid carcinoma is typically rendered when a parathyroid neoplasm shows invasive growth (eg, vascular invasion, lymphatic invasion, perineural invasion, and local gross invasion into the surrounding organs), it is very important to perform immunohistochemical biomarkers of parathyroid carcinoma.^2^ Among these, parafibromin immunohistochemistry not only supports the diagnosis of malignancy in the appropriate morphological setting, but may also provide additional insights into the pathogenesis of these tumors. Specifically, up to 20% of sporadic‐appearing parafibromin‐immunodeficient parathyroid carcinomas can be seen in association with germline *CDC73/HRPT2* mutations. Similarly, global loss of menin (protein encoded by *MEN1*) and p27 (protein encoded by *CDKN1B*) expression can help triaging for MEN1‐ and MEN4‐syndrome‐related manifestations, respectively. The overall morphology with increased mitotic activity and identified angioinvasion in combination with global loss of parafibromin expression in immunohistochemistry confirms the diagnosis of parathyroid carcinoma in this case. Given the absence of germline *CDC73/HRPT2* mutation, the lack of parafibromin expression in the tumor is assumed to occur because of a somatic inactivating *CDC73/HRPT2* mutation with no requirement for the screening of siblings or other family members.

Third, a unique feature of our case was the use of HRpQCT scans before and 1.5 years after surgery. This modality allows for better characterization of bone microarchitecture as well as volumetric density, not possible with standard imaging by DXA and has been used more recently in adults with PHPT.^32^, ^33^ DXA in our case showed a normal *Z*‐score of 1.2 at the lumbar spine (L1 to L4). The lack of areal BMD abnormality detected at the spine can be explained by the otherwise normal density balanced out across a thicker cortex and sparser trabeculae noted on HRpQCT. HRpQCT of both the tibia and radius showed clear abnormalities in structural features involving both cortices (increased cortical porosity) and trabeculae (gross loss of trabeculae), despite showing relatively normal total volumetric bone densities. It is of interest that the distal radius showed greater cortical porosity than the distal tibia and that the porosity at the wrist resolved with treatment, both observations being consistent with what is known of PTH excess and [1‐34] PTH administration in older adults.^34^, ^35^, ^36^

Using HRpQCT we were able to see clear resolution of osteolytic lesions found presurgery at the wrist and ankle (Fig. 3 *A*,*B*). The quality of bone formation within the lesion could also be noted by the concurrent thickening of the cortex and reduction in cortical porosity. In adult patients with PHPT in whom HRpQCT was previously used, osteolytic lesions were not observed.^34^ However, similar structural abnormalities in terms of lost trabeculae and cortical thinning were notable. Previous HRpQCT reports regarding the gain in BMD in adults at the spine, yet nonetheless having multiple vertebral fractures, have explained these findings as a loss of trabecular bone integrity.^37^, ^38^ Our findings are consistent in that BMD was within the normal range, whereas trabecular properties were poor. Stein and colleagues^33^ and Hansen and colleagues^34^ also saw losses in trabecular density, and cortical thickness compared with controls, despite most patients being asymptomatic and not having any areal BMD differences (DXA) even at the distal radius compared with controls. Although Stein and colleagues did not measure cortical porosity, Hansen and colleagues saw a lack of difference in cortical porosity between patients with hyperparathyroidism and controls. This difference could be explained by the fact that the cortex may be more metabolically active in children, especially during pubertal phases such as in the case of our patient, wherein the evolution of cortical porosity is an adaptation mechanism to maintain mechanical strength. In both studies,^33^, ^34^ there was no report of any osteolytic lesions, which may also be explained by this mechanism, but is most likely caused by the higher PTH levels attributed to the carcinoma versus parathyroid adenomas noted in published adult studies.^39^, ^40^, ^41^

It is important to note, however, that the interpretation of HRpQCT bone properties in the context of healthy controls is currently limited for the HRpQCT version 2 of the scanner. The available normative data for children in the literature have only reported results from version 1 scanners,^42^ which use a slightly lower resolution (82‐μm isotropic voxel size) and yield multiple parameters that are not directly measured, but are derived from bone volume fraction and trabecular density. We therefore used several healthy controls (*n* = 5) with same sex and age available from preliminary data of an ongoing healthy cohort study. However, this approach is limited by a small sample size.

In conclusion, although extremely rare, parathyroid carcinoma should be considered in children with severe PHPT. This will ensure appropriate surgical resection and histopathological workup using biomarkers—both of which are crucial for optimizing management and prognosis. HRpQCT provides more detailed insight into the impact on bone than does DXA and shows some features in common with adults, but also others that are specific to the adolescent.

## Disclosure

None of the authors has any conflict of interest to disclose.

## Supporting information


**Table S1.** Published Cases of Children with Parathyroid Carcinoma.
