# Supplementary material for: Severe Primary Hyperparathyroidism Caused by Parathyroid Carcinoma in a 13‐Year‐Old Child; Novel Findings From HRpQCT
Source: JBMR Plus. 2020 Jan 2;4(3):e10324. doi: 10.1002/jbm4.10324 (PMC7059826; doi:10.1002/jbm4.10324)
Supplement: Supplementary file 1 — Table S1. Published Cases of Children with Parathyroid Carcinoma. [file JBM4-4-e10324-s001.docx]

| publication | age (y) and sex | FH of HPT | genetics | PTH (pg/ ml) | pre-operative treatment of hypercalcemia | operative approach | post-operative treatment of hypocalcemia | histopathology | follow up |
| --- | --- | --- | --- | --- | --- | --- | --- | --- | --- |
| Fujimoto 1984 | 16 F | n/a | n/a | 200 | n/a | complete removal of left upper parathyroid gland (2g) | n/a | n/a | at 8y disease free |
| Fujimoto 1984 | 13 F | n/a | n/a | n/a | n/a | removal of right upper pararthyroid gland (15g) | n/a | n/a | at 5 y pulmonary metastasis |
| Young 1984 | 15 M | n/a | n/a | 800 | hyperhydration, mithramycin | en bloc resection (adherence) | none | infiltration of septae | n/a |
| McHenry 1993 | 14 M | + | n/a | 353 | n/a | en bloc resection (infiltration). | n/a | n/a | At 2 y disease free |
| Meier 1999 | 15 M | n/a | n/a | 358 | hyperhydration, furosemide, calcitonin, pamidronate | en bloc resection | iv. Calcium for 9 days | infiltration of the capsula, focal necrosis | At 1y disease free |
| Hamill 2002 | 8 F | + | n/a | 188.6 | n/a | tumor excision | n/a | evidence of capsular and vascular invasion | n/a |
| Fiedler 2009 | 10 M | + | n/a | 300 | n/a | removing mediastinal mass, thymectomy, hemithyroidectomy | Admission for hypocalcemia |  | at 18 months disease free |
| Herrera 2011 | 14 F | - | n/a | 2792 | n/a | right hemithyroidectomy | hungry bone syndrome | infiltration into connecting tissue and vascular system. | at 18 months disease free |
| Kim 2012 | 13 F | - | n/a | 8368 | n/a | en bloc resection (22g) | n/a | capsular, vascular and perineural invasion | at 6 months pulmonal metastasis |
| Vinodh 2012 | 11 M | n/a | n/a |  | n/a | tumor exzision, later hemithyroidectomy | iv Calcium for 6 days | n/a | n/a |
| Rahman 2015 | 10 F | n/a | n/a | 2217 | n/a | n/a | n/a | capsular and vascular invasion | n/a |
| Davidson 2016 | 13 F | - | Germline CDC73**/**HRPT2  mutation | 1068 | hyperhydration, calcitonin, bisphosphonates, cinacalect | en bloc resection +hemithyroidectomy | none | n/a | diffuse metastatic disease including pulmonary metastasis |
| current | 13 M | - | suspected Somatic CDC73**/**HRPT2  mutation | 980 | hyperhydration, calcitonin, bisphosphonates, denosumab | removal of right inferior parathyroid gland | iv Calcium for 3 weeks | vascular invasion, high proliferation, loss of parfibromin immunoreactivity | at 18months disease free |

FH= family history, HPT= Hyperparathyroidism,
